# Supplementary material for: Effect of Malvaviscus arboreus Flower and Leaf Extract on the Functional, Antioxidant, Rheological, Textural, and Sensory Properties of Goat Yogurt
Source: Foods. 2024 Dec 6;13(23):3942. doi: 10.3390/foods13233942 (PMC11640513; doi:10.3390/foods13233942)
Supplement: Supplementary file 1 [file foods-13-03942-s001.zip › foods-3309226-supplementary.pdf]

Supplemental files

Table S1. Phenolic compounds, total flavonoids, and antioxidant activity (FRAP and ABTS) of malvaviscus flower extract (FE) and leaf extract (LE).

Parameters	FE	LE
Phenolic compounds (mg GAE/100g)	7383.02±0.00*	401.89±0,00
Flavonoids (mg CE/100g)	196.04±0.36*	101.88±0.00
FRAP (µmol TEAC/g)	13.27±0.00*	2.02±0.00
ABTS (µmol TEAC/g)	203.28±0.34*	101.8± 0.03

Note: Results are expressed as average (n = 3) ± standard deviation; *Difference between the samples within the same row by Student's t-test ($p < 0.05$).

Table S2. Regression coefficients for the Herschel-Bulkley model adjusted to the experimental data of goat yogurts prepared with different concentrations (1 and 2%) of malvaviscus flower (FE) and leaf (LE) extracts on the 1st day of refrigerated storage (6, 8, 10, and 12 °C).

Formulations	T (°C)	Consistency index (Pa.s^n)	Flow behavior index	R^2 (%)
YC	6	0.06	1.46	99.92
YLE1%		0.01	1.80	99.92
YLE2%		0.08	1.31	99.90
YFE1%		0.02	1.69	99.89
YFE2%		0.03	1.61	99.97
YC	8	0.03	1.60	99.97
YLE1%		0.00	2.00	99.85
YLE2%		0.01	1.79	99.92
YFE1%		0.04	1.60	99.95
YFE2%		0.03	1.55	99.97
YC	10	0.03	1.60	99.90
YLE1%		0.00	2.00	99.86
YLE2%		0.00	1.98	99.86
YFE1%		0.04	1.57	99.97
YFE2%		0.02	1.65	99.95
YC	12	0.01	1.78	99.92
YLE1%		0.01	1.63	99,36
YLE2%		0.00	2.03	99.86
YFE1%		0.02	1.73	99.95
YFE2%		0.02	1.70	99.95

Note: YC: Control goat yogurt; YLE1%: Goat yogurt with 1% addition of LE; YLE2%: Goat yogurt with 2% addition of LE; YFE1%: Goat yogurt with 1% addition of FE; YFE2%: Goat yogurt with 2% addition of FE.

Table S3. Regression coefficients for the Herschel-Bulkley model adjusted to the experimental data of goat yogurts prepared with different concentrations (1 and 2%) of malvaviscus flower (FE) and leaf (LE) extracts on the 14st day of refrigerated storage (6, 8, 10, and 12 °C).

Formulations	T (°C)	Consistency index (Pa.s^n)	Flow behavior index	R^2 (%)
YC	6	0.06	1.43	99.97
YLE1%		0.02	1.55	99.96
YLE2%		0.01	1,74	99.64
YFE1%		0.04	1.53	99.98
YFE2%		0.05	1.45	99.99
YC	8	0.06	1.41	99.99
YLE1%		0.01	1.67	99.89
YLE2%		0.02	1.62	99.93
YFE1%		0.02	1.61	99.92
YFE2%		0.03	1.54	99.97
YC	10	0.05	1.41	99.99
YLE1%		0.01	1,80	99.86
YLE2%		0.00	1.91	99.88
YFE1%		0.02	1.67	99.93
YFE2%		0.04	1.47	99.98
YC	12	0.04	1.46	99.66
YLE1%		0.00	1.89	99.83
YLE2%		0.00	2.13	99.86
YFE1%		0.01	1.72	99.83
YFE2%		0.02	1.58	99.90

Note: YC: Control goat yogurt; YLE1%: Goat yogurt with 1% addition of LE; YLE2%: Goat yogurt with 2% addition of LE; YFE1%: Goat yogurt with 1% addition of FE; YFE2%: Goat yogurt with 2% addition of FE.

Table S4. Regression coefficients for the Herschel-Bulkley model adjusted to the experimental data of goat yogurts prepared with different concentrations (1 and 2%) of malvaviscus flower (FE) and leaf (LE) extracts on the 28st day of refrigerated storage (6, 8, 10, and 12 °C).

Formulations	T (°C)	Consistency index (Pa.s^n)	Fluid behavior index	R^2 (%)
YC	6	0.04	1.45	99.99
YLE1%		0.02	1.63	99.95
YLE2%		0.05	1.36	99.98
YFE1%		0.08	1.38	99.98
YFE2%		0.05	1.43	99.99
YC	8	0.03	1.51	99.97
YLE1%		0.01	1.63	99.90
YLE2%		0.03	1.49	99.95
YFE1%		0.06	1.40	99.98
YFE2%		0.05	1.42	99.98
YC	10	0.03	1.50	99.97
YLE1%		0.01	1,79	99.75
YLE2%		0.01	1.62	99.93
YFE1%		0.04	1.48	99.97
YFE2%		0.04	1.46	99.97
YC	12	0.01	1.65	99.93
YLE1%		0.00	1.94	99.61
YLE2%		0.01	1.75	99.88
YFE1%		0.01	1.73	99.89
YFE2%		0.03	1.51	99.93

Note: YC: Control goat yogurt; YLE1%: Goat yogurt with 1% addition of LE; YLE2%: Goat yogurt with 2% addition of LE; YFE1%: Goat yogurt with 1% addition of FE; YFE2%: Goat yogurt with 2% addition of FE.

Table S5. Cochran's Q test results for the attributes elicited in the CATA test.

Attributes/Samples	YC	YLE1%	YLE2%	YFE1%	YFE2%	p-value
Color opaque	26	31	28	30	23	0.000
Shiny color	37	29	32	38	43	<0.0001
White color	80	0	0	0	0	<0.0001
Pink color	0	0	0	80	80	<0.0001
Green color	0	80	80	0	0	<0.0001
Goat milk aroma	68	34	23	29	25	<0.0001
Herbal aroma	0	25	35	0	0	<0.0001
Floral aroma	0	0	0	17	26	<0.0001
Pleasant aroma	67	39	42	64	65	<0.0001
Unpleasant aroma	0	14	22	0	0	<0.0001
Acidic aroma	5	5	7	3	5	0.122
Adequate texture	41	21	16	46	46	<0.0001
Soft texture	28	26	33	30	46	<0.0001
Viscous	27	42	40	33	37	<0.0001
Creamy	51	22	20	52	47	<0.0001
Aftertaste	8	26	28	16	8	<0.0001
Typical acidity	15	23	30	26	28	<0.0001
Sweet	51	23	21	48	57	<0.0001

Bitter	0	18	30	6	8	<0.0001
Goat milk flavor	50	37	29	42	37	<0.0001
Intense goat milk flavor	42	32	30	7	16	<0.0001
Floral flavour	0	0	0	31	28	<0.0001
Herbal flavour	0	44	62	0	0	<0.0001
Characteristic yogurt flavor	47	14	15	49	62	<0.0001

Note: Results expressed in frequency. (n=80); YC: Control goat yogurt; YLE1%: Goat yogurt with 1% addition of LE; YLE2%: Goat yogurt with 2% addition of LE; YFE1%: Goat yogurt with 1% addition of FE; YFE2%: Goat yogurt with 2% addition of FE.

Table S6. Multiple comparison analysis of the elicited atributes.

Attributes	YC	YLE1%	YLE2%	YFE1%	YFE2%
Opaque	0.325 (ab)	0.388 (b)	0.350 (ab)	0.375 (b)	0.287 (a)
Shiny	0.463 (bc)	0.362 (a)	0.400 (ab)	0.475 (bc)	0.537 (c)
White	1 (b)	0 (a)	0 (a)	0 (a)	0 (a)
Pink	0 (a)	0 (a)	0 (a)	1 (b)	1 (b)
Green	0 (a)	1 (b)	1 (b)	0 (a)	0 (a)
Goat milk aroma	0.850 (b)	0.425 (a)	0.287 (a)	0.362 (a)	0.312 (a)
Herbal aroma	0 (a)	0.312 (b)	0.438 (b)	0 (a)	0 (a)
Floral aroma	0 (a)	0 (a)	0 (a)	0.212 (b)	0.325 (b)
Pleasant aroma	0.838 (b)	0.487 (a)	0.525 (a)	0.800 (b)	0.812 (b)
Unpleasant aroma	0 (a)	0.175 (b)	0.275 (b)	0 (a)	0 (a)
Acidic aroma	0.062 (a)	0.062 (a)	0.087 (a)	0.037 (a)	0.062 (a)
Adequate texture	0.512 (b)	0.263 (a)	0.200 (a)	0.575 (b)	0.575 (b)
Soft texture	0.350 (a)	0.325 (a)	0.412 (a)	0.375 (a)	0.575 (b)
Viscous	0.338 (a)	0.525 (c)	0.500 (bc)	0.412 (ab)	0.463 (bc)
Creamy	0.637 (b)	0.275 (a)	0.250 (a)	0.650 (b)	0.588 (b)
Aftertaste	0.100 (a)	0.325 (b)	0.350 (b)	0.200 (a)	0.100 (a)
Typical acidity	0.188 (a)	0.287 (b)	0.375 (b)	0.325 (b)	0.350 (b)
Sweet	0.637 (b)	0.287 (a)	0.263 (a)	0.600 (b)	0.713 (b)
Bitter	0 (a)	0.225 (b)	0.375 (c)	0.075 (a)	0.100 (ab)
Goat milk flavor	0.625 (c)	0.463 (ab)	0.362 (a)	0.525 (bc)	0.463 (ab)

Intense goat milk flavor	0.525 (c)	0.400 (bc)	0.375 (b)	0.087 (a)	0.200 (a)
Floral flavour	0 (a)	0 (a)	0 (a)	0.388 (b)	0.350 (b)
Herbal flavour	0 (a)	0.550 (b)	0.775 (c)	0 (a)	0 (a)
Characteristic yogurt flavor	0.588 (b)	0.175 (a)	0.188 (a)	0.613 (bc)	0.775 (c)

Note: The Critical Difference (Sheskin) procedure was utilized (n=80); Results obtained from binary frequency data; Different letters indicate statistical differences at the 5% level among samples; YC: Control goat yogurt; YLE1%: Goat yogurt with 1% addition of LE; YLE2%: Goat yogurt with 2% addition of LE; YFE1%: Goat yogurt with 1% addition of FE; YFE2%: Goat yogurt with 2% addition of FE.

Table S7. Results of the independence test of attributes (CATA test), performed for each formulation.

Formulations	p-values
YC	<0.0001
YLE1%	<0.0001
YLE2%	<0.0001
YFE1%	<0.0001
YFE2%	<0.0001

Note: Chi-square test of independence; For at least one combination of a product with an attribute, the attribute was marked or unmarked by all assessors, and the independence test could not be calculated; YC: Control goat yogurt; YLE1%: Goat yogurt with 1% addition of LE; YLE2%: Goat yogurt with 2% addition of LE; YFE1%: Goat yogurt with 1% addition of FE; YFE2%: Goat yogurt with 2% addition of FE.

Figure S1. Rheograms of goat yogurts prepared with different concentrations (1 and 2%) of malvaviscus flower (FE) and leaf (LE) extracts at different temperatures (6, 8, 10, and 12 °C) and storage times (1, 14, and 28 days) with the Herschel-Bulkley model adjusted to the experimental data.

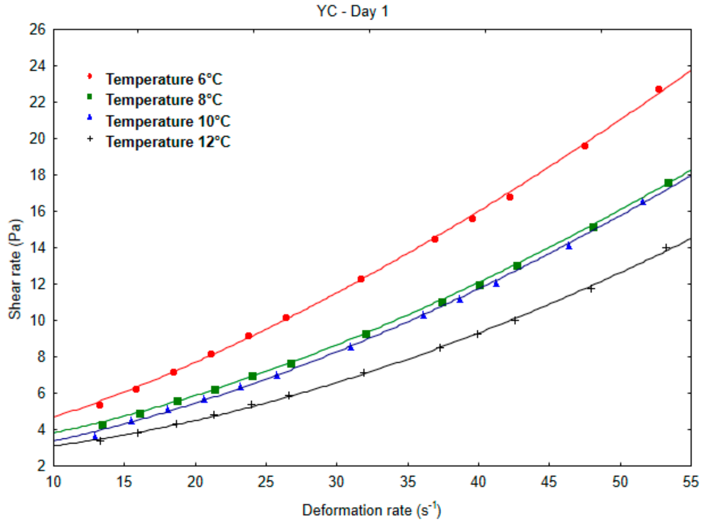

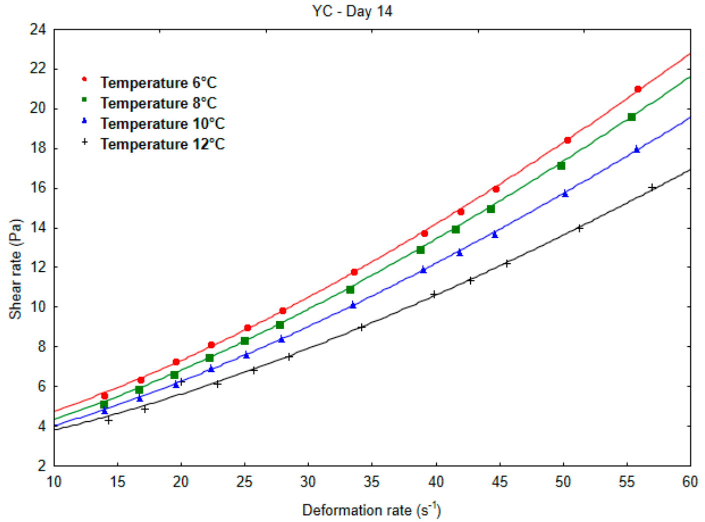

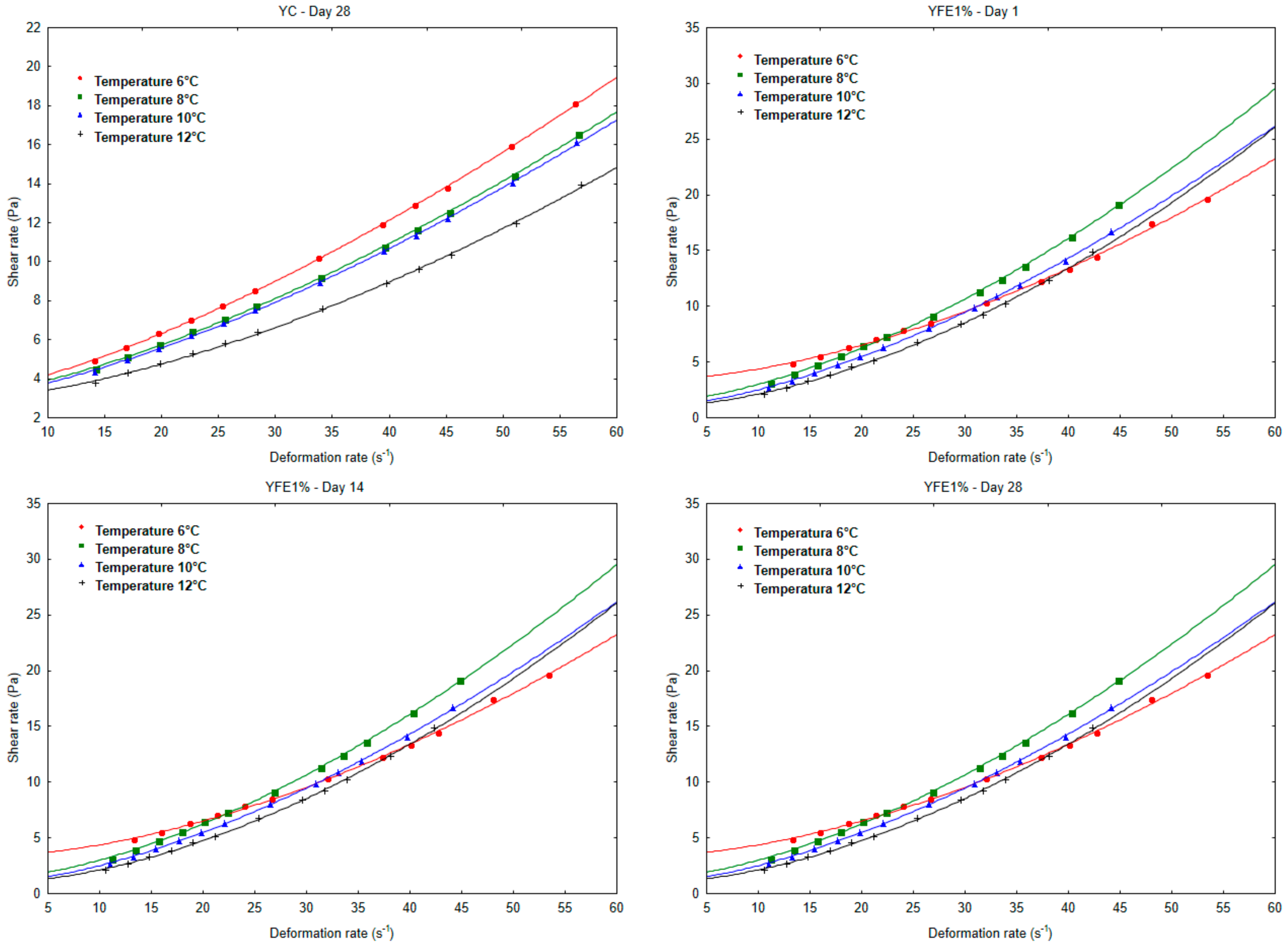
YC - Day 28
Temperature 6°C
Temperature 8°C
Temperature 10°C
Temperature 12°C
Shear rate (Pa)
Deformation rate (s^{-1})
YFE1% - Day 1
Temperature 6°C
Temperature 8°C
Temperature 10°C
Temperature 12°C
Shear rate (Pa)
Deformation rate (s^{-1})
YFE1% - Day 14
Temperature 6°C
Temperature 8°C
Temperature 10°C
Temperature 12°C
Shear rate (Pa)
Deformation rate (s^{-1})
YFE1% - Day 28
Temperatura 6°C
Temperatura 8°C
Temperatura 10°C
Temperatura 12°C
Shear rate (Pa)
Deformation rate (s^{-1})

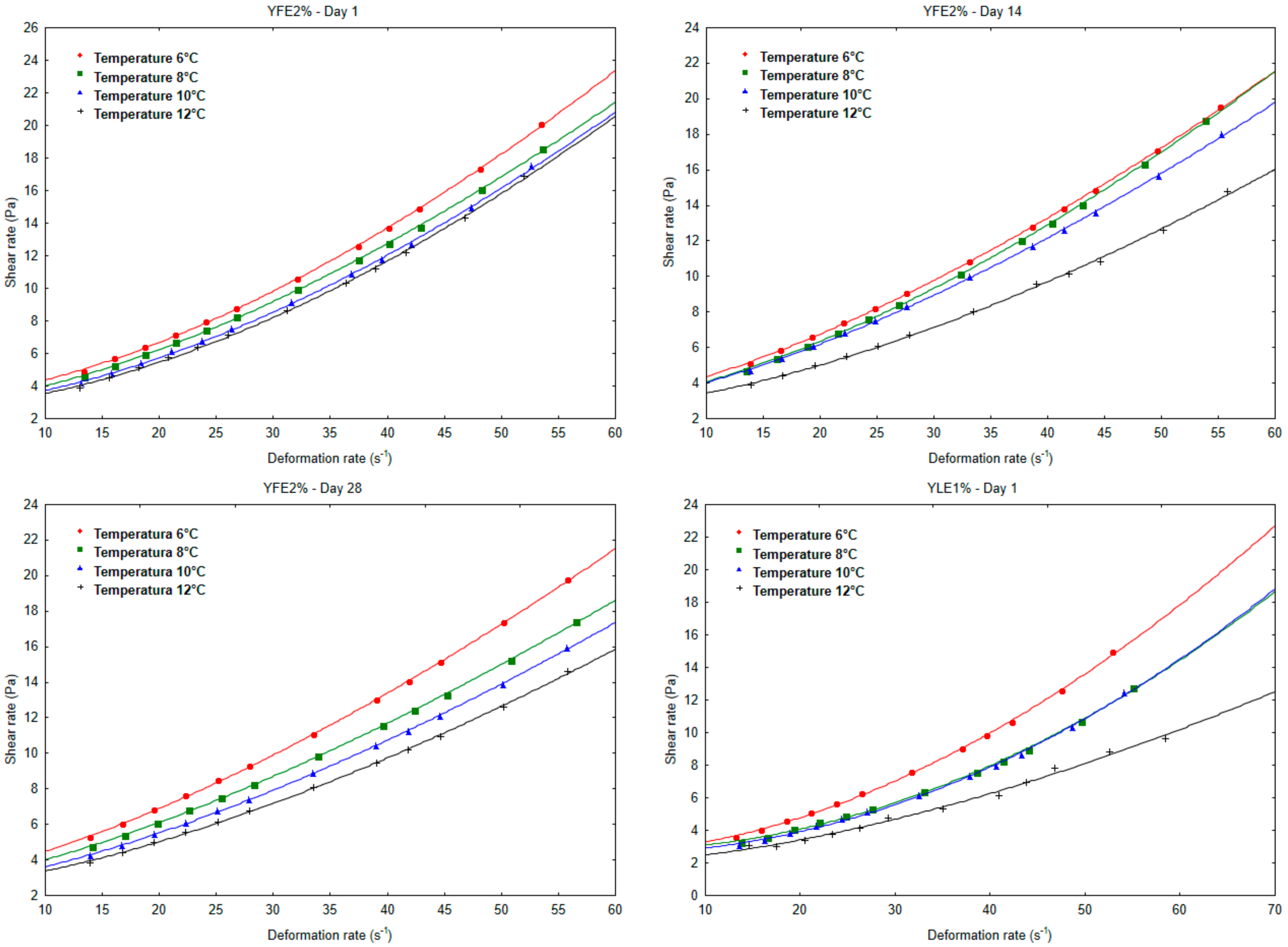
YFE2% - Day 1
Temperature 6°C
Temperature 8°C
Temperature 10°C
Temperature 12°C
Shear rate (Pa)
Deformation rate (s^{-1})
YFE2% - Day 14
Temperature 6°C
Temperature 8°C
Temperature 10°C
Temperature 12°C
Shear rate (Pa)
Deformation rate (s^{-1})
YFE2% - Day 28
Temperatura 6°C
Temperatura 8°C
Temperatura 10°C
Temperatura 12°C
Shear rate (Pa)
Deformation rate (s^{-1})
YLE1% - Day 1
Temperature 6°C
Temperature 8°C
Temperature 10°C
Temperature 12°C
Shear rate (Pa)
Deformation rate (s^{-1})

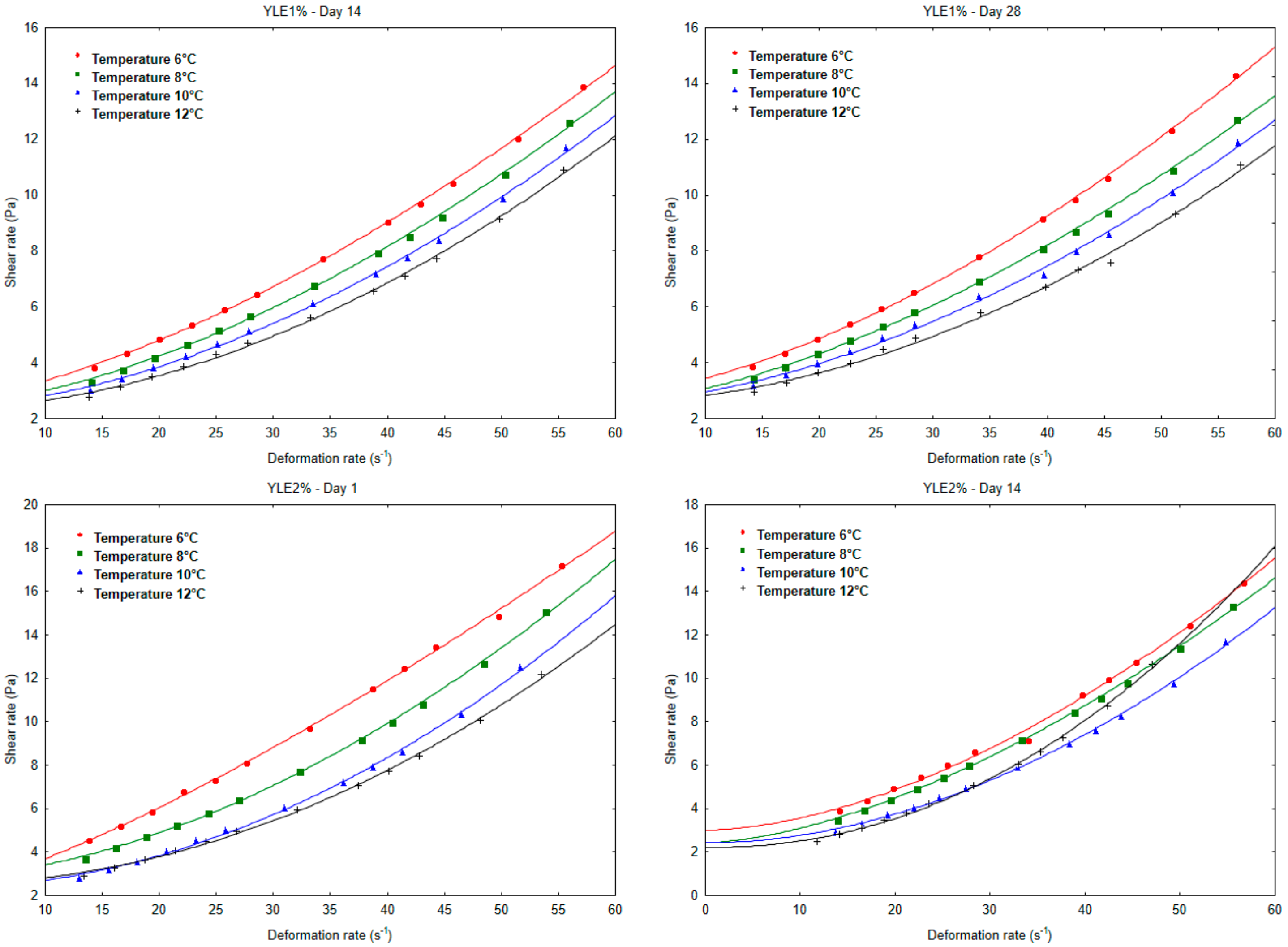
YLE1% - Day 14
YLE1% - Day 28
YLE2% - Day 1
YLE2% - Day 14
Temperature 6°C
Temperature 8°C
Temperature 10°C
Temperature 12°C
Shear rate (Pa)
Deformation rate (s^{-1})

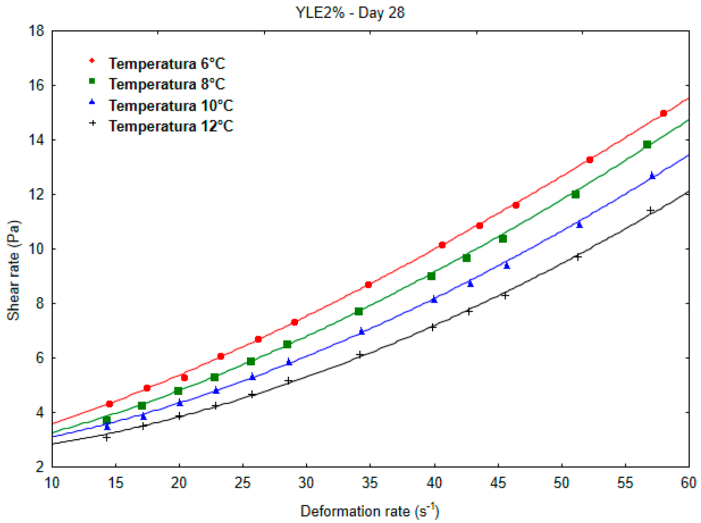
YLE2% - Day 28
Temperatura 6°C
Temperatura 8°C
Temperatura 10°C
Temperatura 12°C
Shear rate (Pa)
Deformation rate (s-1)
18
16
14
12
10
8
6
4
2
10
15
20
25
30
35
40
45
50
55
60
